# Slow intrinsic oscillations in the ventrolateral preoptic nucleus

**DOI:** 10.1016/j.isci.2026.116477

**Published:** 2026-07-03

**Authors:** Quentin Perrenoud, Jérôme Ribot, Hélène Geoffroy, Thierry Gallopin, Nathalie Rouach, Armelle Rancillac

**Affiliations:** 1 Institut de biologie de l'Ecole normale supérieure (IBENS), Ecole normale supérieure, CNRS, INSERM, PSL Research University, France; 2 Neuroglial Interactions in Cerebral Physiology and Pathologies, CIRB, Collège de France, CNRS UMR 7241, INSERM U1050, PSL University, PSL-Neuro, Paris 75005, France; 3 Brain Plasticity Unit, ESPCI-Paris, PSL Research University, Paris 75005, France

**Keywords:** Biological sciences

## Abstract

The ventrolateral preoptic nucleus (VLPO) promotes non-rapid eye movement (NREM) sleep. While VLPO neurons often display low-threshold spikes (LTSs), a feature that supports rhythmic activity, rhythmic bursting has never been reported in these neurons. Here, we report that ∼12% of VLPO neurons in a large *ex vivo* patch-clamp dataset exhibit spontaneous rhythmic bursting. This pattern occurred in putative sleep-promoting neurons (inhibited by noradrenaline, NA), and in wake-active neurons (excited by NA). Unsupervised clustering of 24 bursting neurons using burst parameters, electrophysiological, and morphological features revealed three groups: one putative sleep-promoting subtype and two wake-active subtypes with fast and slow bursting. Strikingly, membrane potential oscillations persisted in tetrodotoxin (TTX), indicating an intrinsic mechanism. Bursts of inhibitory inputs were also recorded on sleep-promoting neurons. These results suggest that intrinsic rhythmic bursting may propagate to the local network, with functional relevance for sleep regulation.

## Highlights

• A subset of VLPO neurons exhibits spontaneous rhythmic bursting
• Bursting VLPO neurons can be inhibited or activated by noradrenaline
• Unsupervised clustering revealed three clusters of bursting neurons
• Bursting is intrinsically generated and may be tuned by extrinsic mechanisms

## Graphical abstract

**Figure:**
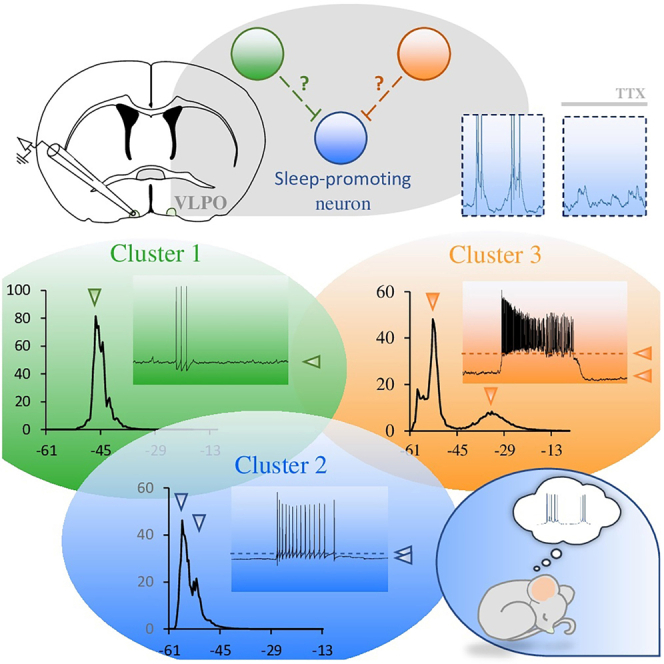

## Introduction

Sleep is a fundamental and ubiquitous behavior across the animal kingdom, but its function and mechanism remain actively debated.^1^ In mammals, sleep alternates between two distinct physiological states: non-rapid eye movement (NREM) sleep and rapid eye movement (REM) sleep. The induction and maintenance are regulated by complex networks of interconnected brain nuclei spanning the forebrain, midbrain, and hindbrain.^2^^,^^3^^,^^4^^,^^5^ Among these, the ventrolateral preoptic nucleus (VLPO), a diffuse region of the ventral hypothalamus, constitutes a key promoter of NREM sleep.^6^^,^^7^ However, the circuit mechanisms underlying its function are not yet fully understood.

The VLPO receives convergent inputs from several wake-promoting regions, including the locus coeruleus (LC), the tuberomammillary nucleus (TMN), and the lateral hypothalamus (LH), which respectively release noradrenaline (NA), histamine, and orexin. It also integrates cholinergic, serotonergic, glutamatergic, and GABAergic projections arising from various brain sites.^8^ Among the heterogeneous neuronal populations in the VLPO,^9^^,^^10^^,^^11^^,^^12^ a key subset, referred to as sleep-promoting neurons, is directly inhibited by NA^9^^,^^13^ and indirectly inhibited by histamine^14^^,^^15^^,^^16^ and orexin.^17^^,^^18^ These neurons, in turn, inhibit wake-promoting nuclei via GABAergic and galaninergic projections,^14^^,^^19^^,^^20^ thereby contributing to sleep onset and maintenance.

A commonly proposed model suggests that the mutual inhibition between wake-promoting centers and sleep-promoting VLPO neurons creates a bistable system that toggles between sleep and wakefulness.^21^ Nevertheless, this model does not fully account for several observations. Notably, simple mutually inhibitory circuits typically generate abrupt and stochastic state transitions, whereas natural transitions between wakefulness, NREM, and REM sleep are gradual and well-ordered, suggesting the presence of complex regulatory mechanisms.^8^^,^^10^^,^^20^^,^^22^^,^^23^^,^^24^ This highlights a need for further research to understand the mechanisms governing the patterning of activity in VLPO neurons.

Neural types other than sleep-promoting neurons exist in the VLPO.^9^^,^^10^^,^^11^^,^^12^ These neurons are predominantly GABAergic and excited by NA, suggesting they may exert inhibitory control over sleep-promoting neurons during wakefulness.^15^^,^^16^^,^^18^ However, the VLPO is neurochemically heterogeneous, and glutamatergic wake-promoting neurons have also been described, including populations of Wake/REM ON activity.^25^^,^^26^^,^^27^ Sleep-promoting VLPO neurons can generate low-threshold spike (LTS),^9^^,^^10^^,^^12^^,^^23^^,^^24^ a well-described rebound event mediated by low-threshold (T-type) Ca^2+^ conductance that is classically recruited upon release from sufficiently strong hyperpolarization, producing a depolarizing rebound that can support burst firing and rhythmicity.^28^^,^^29^ Yet, whether VLPO neurons exhibit such oscillatory activity, and how it contributes to their role in sleep regulation, remains unknown.

Here, we report that a subset of VLPO neurons exhibits intrinsically generated, slow rhythmic bursting activity that corresponds to the inverse of the oscillatory cycle period. Among a dataset of 393 *ex vivo* recordings (cell-attached and whole-cell), we identified 37 neurons displaying spontaneous rhythmic bursting. Twelve of them were inhibited by NA, a hallmark of sleep-promoting identity. We further characterized the electrophysiological and morphological properties of 24 bursting neurons recorded in the whole-cell configuration. Using unsupervised clustering, we identified three distinct clusters: two groups of NA-activated neurons exhibiting rapid and slow bursting patterns, respectively (i.e., short and long cycle periods), and a third group of NA-inhibited (putative sleep-promoting) neurons displaying intermediate bursting cycle period. In a subset of neurons, the application of tetrodotoxin (TTX) did not affect membrane potential oscillations, indicating that the underlying rhythmic activity was maintained independently of sodium-dependent spike generation. These observations reveal that VLPO neurons can intrinsically generate rhythmic activity, which may influence sleep-wake network dynamics.

## Results

### Spontaneous rhythmic bursting in VLPO neurons

While investigating the properties of VLPO neurons with patch-clamp recording in juvenile slices, we occasionally observed spontaneous, rhythmic activity in the form of a regular burst of action potentials (APs). We thus explored the properties of bursting VLPO neurons.

**Figure 1:**
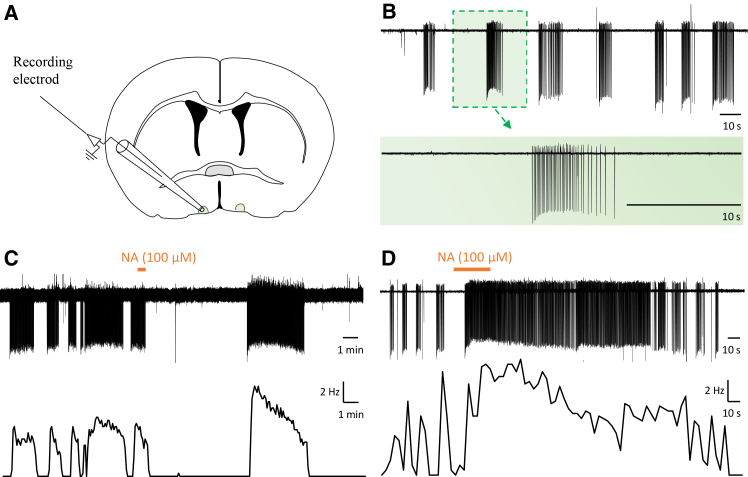
Recordings of bursting neurons in a loose-patch configuration in the VLPO (A) Localization of the VLPO and the recording electrode on a coronal slice (around bregma 0.18). (B) Typical recording of rhythmic sequences of discharge in loose cell-attach configuration within the VLPO (upper trace). An expansion of one bursting sequence is represented in the lower panel. (C and D) NA (10 μM) robustly decreases (C) or increases (D) the firing frequency of discharge. Discharge rates are represented in the lower trace with a bin of 5 s.

Across 393 recordings in our previously published patch-clamp datasets,^11^^,^^12^^,^^23^^,^^30^ we identified 49 spontaneously bursting neurons, accounting for 12.5% of the total (Figure 1). Here, we restricted our analyses to bursting recordings of sufficient duration to allow quantitative analyses, yielding a final dataset of 37 VLPO neurons recorded in the loose patch (*n* = 18) and/or whole-cell configuration (*n* = 24). For five neurons, recordings were obtained sequentially in both configurations, and in all instances, the shift to a whole-cell configuration did not affect bursting.

We first analyzed neurons recorded in the loose-patch configuration. As VLPO neurons are classically classified by their response to NA, with NA-induced hyperpolarization defining NA(−) (putative sleep-promoting) neurons and depolarization defining NA(+) neurons,^9^ we assessed NA responsiveness in this sample. We found NA inhibited neurons, 7 of 18 (Figure 1C) and excited neurons, 11 of 18 (Figure 1D). A detailed comparison of their bursting features revealed that NA(−) exhibited a longer burst duration and a lower burst frequency compared to NA(+) neurons (Table 1), while other metrics showed no statistical differences, indicating that rhythmic bursting is expressed across NA-defined VLPO We next analyzed VLPO neurons recorded in the whole-cell configuration to explore how bursting relates to intrinsic membrane potential dynamics and neuronal type. Of of 24 neurons, 5 were inhibited in response to NA application, thus corresponding to putative sleep-promoting neurons. To further explore whether distinct subpopulations of bursting neurons were present in our sample, we examined intrinsic electrophysiological properties estimated immediately after achieving intracellular access as well as morphological characteristics estimated from infrared pictures of somata taken before recordings and 3-dimensional reconstructions of dendritic arbors obtained after intracellular biocytin filling and subsequent Neurolucida reconstructions (Materials and Methods).

**Table 1:** Bursting features of NA(−) and NA(+) neurons

	NA (−) (*n* = 7)	NA (+) (*n* = 11)
Burst Duration (s)	**3.6 ± 0.3**	**1.7 ± 0.1∗**
Burst Frequency (Hz)	**0.1 ± 0**	**0.3 ± 0∗**
Interbust Intervals (s)	7.8 ± 0.6	5.6 ± 0.3
Mean AP number/Bust	16.6 ± 1.8	14.6 ± 1.1
Mean Intra Bursts Freq. (Hz)	5.3 ± 0.5	13 ± 1.1
Mean Inter Burst Freq. (Hz)	1.5 ± 0.2	0.6 ± 0
Mean AP Freq. (Hz)	2.7 ± 0.3	5 ± 0.5

Both NA(−) and NA(+) VLPO neurons exhibit burst firing activity with broadly similar properties, except for their burst duration and burst frequency, which differ significantly depending on their response to NA application. ∗*p* < 0.05, Mann-Whitney test.

### Unsupervised clustering identifies three subtypes of bursting neurons

**Figure 2:**
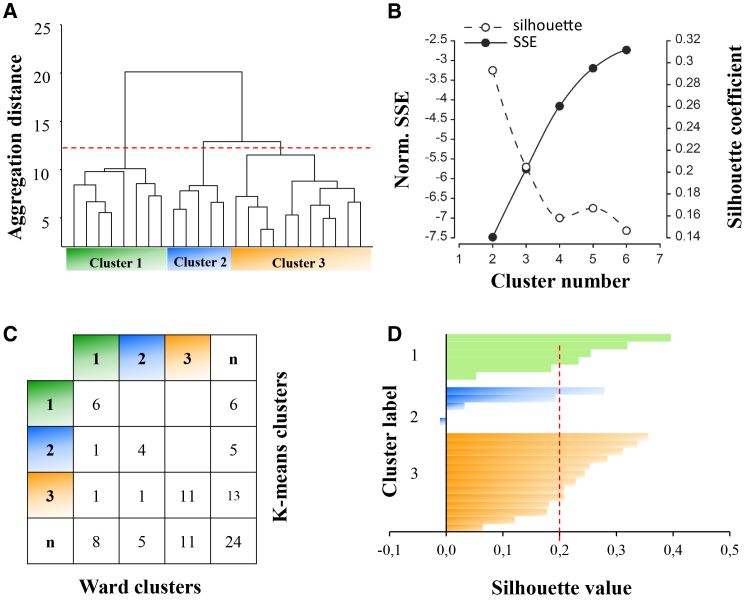
Unsupervised clustering of bursting VLPO neurons (A) Ward’s clustering of 24 bursting neurons. Individual cells are represented along the x axis. The y axis represents the average within-cluster linkage distance in a space of 34 electrophysiological and morphological characteristics. Three clusters: 1 (in green), 2 (in blue), and 3 (in orange) were identified. (B) Normalized SSE, and silhouette coefficients versus number of clusters. (C) Clusters generated by Ward’s method in (A) were corrected using the clustering output generated by the *K-means* algorithm. (D) The silhouette analysis was performed to assess the quality of the clustering (mean value of 0.203; red dashed line).

A feature set of 34 parameters encompassing burst dynamics, as well as electrophysiological and morphological properties, was selected for unsupervised clustering (Materials and Methods). Ward’s method^31^ was first applied to build a dendrogram representing the similarity between bursting neurons (Figure 2A). Clusters generated by Ward’s method were corrected by applying the k-means algorithm to ensure that clusters occupied non-overlapping regions of the feature space.^12^^,^^32^ Partitions in 2–6 clusters were considered (Figure S1).^33^ The normalized total within-cluster sum of squared errors (SSE; STAR Methods) presented a minimum for 2 clusters and increased progressively for larger cluster numbers (Figure 2B). Likewise, the average silhouette value presented a maximum at 2 clusters and decreased for progressively finer partitions (Figure 2B), indicating a clear partition into two major groups of neurons (Figure S1A). However, when examining partitions into 3 clusters, we noticed that one of these groups split into NA- and NA + subgroups (respectively in cluster 2 and cluster 3, Figures 1A and S1B). We thus decided to retain a partition into 3 clusters. When considering 3 groups, the k-means algorithm reassigned a total of 3 neurons from Ward’s dendrogram (Figure 2C), resulting in a partition having a mean silhouette value of 0.205 (Figure 2D).

While our feature space captures a comprehensive array of neural properties, its large number of parameters (34) relative to a low number of observations (24 neurons) could potentially result in non-meaningful separation between clusters. To control for this, we examined the robustness of our clustering when applying dimensionality reduction through principal component analysis (PCA, Figure S2A). Clustering was performed on several principal components (PCs) ranging from 9 to 2. Grouping was unchanged when considering 9 PCs. Considering a lower number of PCs tended to shuffle neurons between cluster 2 and 3, confirming the proximity between these groups of neurons. Finally, grouping based on 2 or 3 PCs resulted in minimal reassignment of neurons (Figure S2A). This indicates that our grouping is not artificially introduced by the high number of parameters considered but reflects a relatively low-dimensional variance structure.

To further understand the influence of parameters on the grouping of neurons, we repeated clustering while excluding (Figure S2B) or selectively including (Figure S2C) groups of functionally related parameters (STAR Methods). Cluster assignment remained mostly conserved when removing parameter groups. Two notable exceptions were passive electrophysiological parameters and noradrenalin responsiveness, whose removal disrupted the assignment of neurons between clusters 2 and 3 (Figure S2B). This confirms a proximity between these clusters and confirms that they are differentiated by their response to NA. Conversely, clustering based solely on selected parameter groups resulted in major reshuffling of cluster identity (Figure S2C). This indicates that selected groups of parameters can’t capture our clustering structure and that this structure emerges from the combination of these parameters.

### Cluster-specific pharmacological, electrophysiological, morphological, and activity-dependent properties of bursting neurons

**Figure 3:**
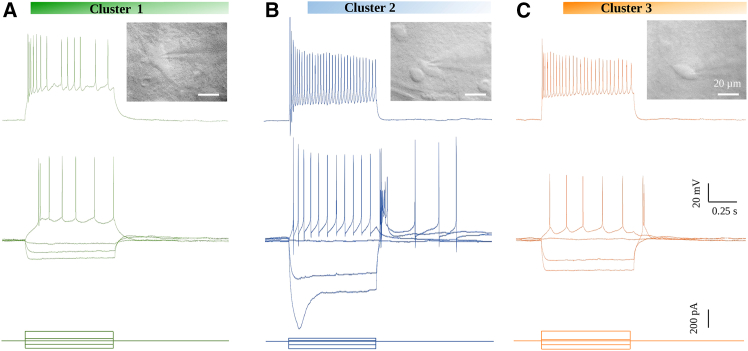
Passive membrane properties and firing properties of the bursting VLPO neurons (A) Typical electrophysiological behavior of neurons from cluster 1 (green, *top*). Insert: Infrared image of the recorded neuron in whole-cell configuration. A strong depolarizing current (*top trace*) evoked a high and sustained firing. Responses to −100, −50, 0 pA, and threshold current pulses (*middle and bottom traces*). (B and C), same as in A, but for a neuron from cluster 2 (blue) and cluster 3 (yellow).

**Figure 4:**
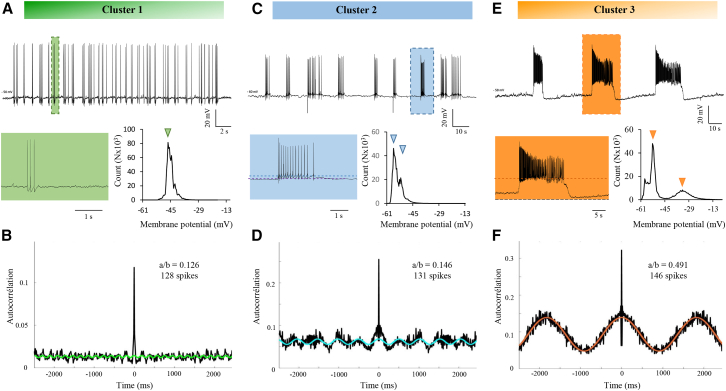
Busting properties and passive membrane features (A) Whole-cell recording of spontaneous bursting firing at the resting membrane potential (RMP) of −50 mV (*top*). Inset (green rectangle): magnified view of a single burst. Bottom right: histogram of membrane potential values for cluster 1 neurons. Membrane potential was continuously sampled from the raw whole-cell recording, and all data points were included. The preferred membrane potential is indicated by an arrow. (B) Spiking auto-correlogram reveals a periodicity. (C–F), same as in A-B but for representative neurons from clusters 2 (blue, RMP -60 mV) and cluster 3 (orange, RMP -50 mV), respectively.

**Figure 5:**
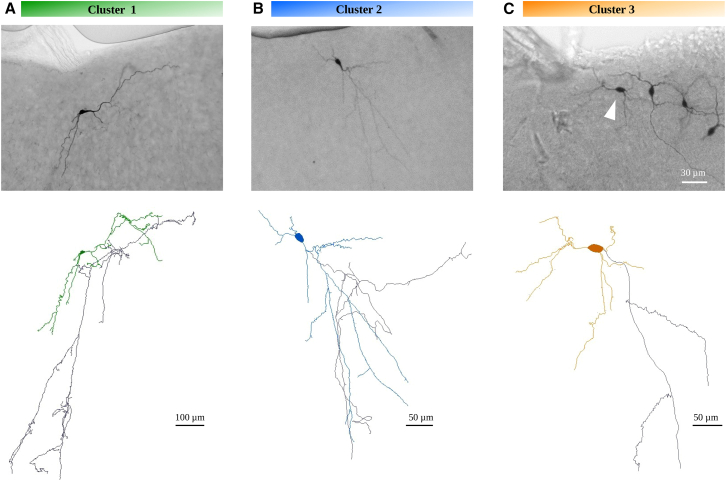
Neurolucida reconstructions of typical bursting neurons from the three clusters (A) Representative cluster 1 neuron: epifluorescence image of a biocytin-filled bursting VLPO neuron (*top*) and its Neurolucida reconstruction (*bottom*). Dendrites and soma are shown in green; the axon in gray. Scalebar: 30 μm. (B) Same as in (A) for a cluster 2 neuron. (C) Same as in (A) for a cluster 3 neuron. The white ar indicates the recorded, biocytin-filled neuron.

Neurons in cluster 1 (*n* = 6/24) were all excited by NA (Figure S3) and rarely displayed LTS or rebound from hyperpolarization (Table 2), consistent with a non-sleep-promoting neuron phenotype. These neurons had relatively low membrane resistance, high rheobases (Table 2), and frequently displayed a stuttering firing pattern (Figures 3A and S3A). Bursting in these neurons was not accompanied by marked variations in the membrane potential called UP and DOWN phases (Figure 4A and Table 3). Bursts of APs were typically short and alternated rapidly with epochs of silence, recurring intrinsically with a periodicity of once every ∼200 ms (Figure 4B and Table 3). Morphologically, cluster 1 neurons present large fusiform somata and well-developed dendritic arbors that extend significantly farther than in other clusters (Figure 5 and Table 4).

**Table 2:** Electrophysiological properties of bursting VLPO neurons

	**1** (*n* = 6)	**2** (*n* = 5)	**3** (*n* = 13)
(1) RMP (mV)	−51.4 ± 1.4	−46.7 ± 2.4	−48.4 ± 1.5
		–	
(2) R_m_ (MΩ)	356.9 ± 116.5	900 ± 241.6	570.6 ± 51.6
		*p* < 0.08	
(3) **t**_**IC**_ (ms)	21.1 ± 4.3	29 ± 3.1	35.5 ± 3.7
		–	
(4) C_m_ (pF)	71.1 ± 9.1	45 ± 13.9	64.3 ± 4.9
		–	
(5) R_hyp_ (MΩ)	285.8 ± 85.7	695 ± 186.7	384.4 ± 43.2
		*p* < 0.09	
(6) R_sag_ (MΩ)	220.7 ± 61.5	458.5 ± 116	310.7 ± 29.2
		–	
(7) ΔG_sag_ (ratio)	78.5 ± 6.5	66.5 ± 5.7	83 ± 2.6
		–	
(8) **Rebond** (%)	**0**	**80**	**92.3**
		**∗∗∗**	
(9) **LTS** (%)	**16.7**	**80**	**92.3**
		**∗∗**	
(10) **Rheobase** (pA)	**38.3** ± **6**	**16** ± **2.4**	**16.2** ± **2.4**
		**∗∗**	
(11) 1^st^ spike latency (ms)	113.3 ± 17.4	78.5 ± 16.1	98.1 ± 16.6
		–	
(12) m_threshold_ (Hz/s)	−29.5 ± 25.9	−43.9 ± 16.3	−62.8 ± 18.8
		–	
(13) F_threshold_ (Hz)	9.4 ± 3.3	35.7 ± 16.5	33 ± 6.9
		–	
(14) A_sat_ (Hz)	112.7 ± 17.3	69.3 ± 29.8	65.6 ± 8.8
		–	
(15) t_sat_ (ms)	28.8 ± 8.5	40.9 ± 12.6	34.2 ± 4.6
		–	
(16) F__offset_ (Hz)	92.6 ± 18.5	45.9 ± 3.7	46.3 ± 5.6
		–	
(17) m_sat_ (Hz/s)	−18.8 ± 9.9	−0.5 ± 6.5	−15.4 ± 3.1
		–	
(18) A1 (mV)	94.1 ± 12.3	91.6 ± 10.8	93.8 ± 7.2
		–	
(19) D1 (ms)	1 ± 0.3	1.2 ± 0.2	1.1 ± 0.1
		–	
(20) **AHP**_**max**_ (mV)	**−5.1** ± **1.3**	**−24.7** ± **5.2**	**−10.4** ± **3.1**
		∗	
(21) tAHP_max_ (ms)	24.2 ± 6.6	10.5 ± 7.2	12.3 ± 4.7
		–	
(22) ADP (mV)	5.8 ± 3.2	2.8 ± 2.8	0.7 ± 0.4
		–	
(23) tADP (ms)	2.7 ± 0.8	0.3 ± 0.3	1.9 ± 1
		–	
(24) A2 (mV)	90 ± 12	81.7 ± 11.8	83.6 ± 6.9
		–	
(25) D2 (ms)	1.1 ± 0.3	1.4 ± 0.3	1.3 ± 0.1
		–	
(26) Var A (%)	4.6 ± 2.1	12.1 ± 3.2	11.2 ± 1.6
		–	
(27) Var D (%)	−3.4 ± 1.1	−10.6 ± 5.1	−9 ± 2.3
		–	
(28) **NA** (%)	**100 (+)**	**80 (−)**	**92.3 (+)**
		**∗∗**	

Abbreviations are defined in the Electrophysiological section of the STAR Methods. Kruskal–Wallis test, followed by a Dunn’s post hoc test. Bold type font indicates significance; ∗, ∗∗ and ∗∗∗: significant with p < 0.05, p < 0.01 and p < 0.001 respectively.

**Table 3:** Bursting properties of VLPO neurons

	1 (*n* = 6)	2 (*n* = 5)	3 (*n* = 13)
(1) Depolarized phase (mV)	−46.1 ± 2.8	−46.5 ± 3.1	−46.1 ± 1.6
		–	
(2) Hyperpolarized phase (mV)	−46.8 ± 2.9	−51.1 ± 4.4	−54.8 ± 1.2
		–	
(3) **Delta** (mV)	**−0.7** ± **0.4**	**−4.6** ± **2.1**	**−8.7** ± **1.6**
		**∗**	
(4) a/b	0.3 ± 0.1	0.2 ± 0	0.5 ± 0.1
		–	
(5) **Period T** (s)	**1.9** ± **0.7**	**13.2** ± **5.3**	**18.9** ± **3.3**
		**∗∗**	
(6) **Rise Time** (ms)	**12.7** ± **5.6**	**128.3** ± **55.2**	**143.5** ± **27.1**
		**∗∗**	
(7) **Decay** Time (ms)	**27.1** ± **15.9**	**221.5** ± **73.8**	**378.8** ± **77.8**
		**∗∗**	
(8) **Burst duration** (s)	**0.7** ± **0.3**	**1.7** ± **0.1**	**4.8** ± **1**
		**∗**	
(9) Burst Frequency (Hz)	0.1 ± 0.1	0.2 ± 0.1	0.1 ± 0
		–	
(10) **Nb of AP/burst**	**4.0** ± **1.2**	**7.4** ± **1.3**	**24.3** ± **5.5**
		∗∗	
(11) Intra Burst Frequency (Hz)	6.3 ± 2.1	4.8 ± 0.3	5.6 ± 0.6
		–	
(12) **InterBurst Frequency** (Hz)	**0.4** ± **0.1**	**0.5** ± **0.2**	**0.1** ± **0**
		**∗∗**	
(13) Mean AP frequency (Hz)	1.6 ± 0.5	1.8 ± 0.3	1.8 ± 0.6
		–	
(14) Interbuste interval (ms)	856.2 ± 234.7	639.1 ± 183.8	788.7 ± 170.4

Kruskal–Wallis test, followed by a Dunn’s post hoc test. Bold type font indicates significance; ∗ and ∗∗: significant with p < 0.05 and p < 0.01 respectively.

**Table 4:** Somatic properties of bursting neurons on infrared images before patch-clamp recordings

	1 (*n* = 6)	2 (*n* = 5)	3 (*n* = 13)
(1) Cell body area (μm^2^)	253.1 ± 52.8	181.5 ± 32.5	193.9 ± 13.2
		–	
(2) Perimeter (μm)	68.6 ± 9.3	53.8 ± 5.5	56.9 ± 3.3
		–	
(3) Form factor	0.7 ± 0.1	0.8 ± 0	0.8 ± 0
		–	
(4) Feret max (μm)	26.1 ± 5.2	21 ± 2.6	21.8 ± 1.4
		–	
(5) Feret min (μm)	13.1 ± 1.5	11.8 ± 1.3	13.2 ± 0.6
		–	
(6) Aspect ratio	2 ± 0.4	1.9 ± 0.3	1.6 ± 0.1
		–	
(7) Solidity	1 ± 0	1 ± 0	0.9 ± 0
		–	
(8) Convexity	0.9 ± 0	1 ± 0	1 ± 0
		–	
(9) Roundness	1.5 ± 0.2	1.3 ± 0.1	1.3 ± 0.1
		–	
(10) Compactness	1.2 ± 0.1	1.1 ± 0	1.2 ± 0
		–	

n, number of cells; Kruskal–Wallis test.

Cluster 2 neurons (*n* = 5/24) were mostly inhibited by NA (Figure S2) and displayed prominent LTS and rebound following hyperpolarization (Table 2), consistent with a sleep-promoting phenotype. Accordingly, these neurons presented a high membrane resistance, a pronounced delayed rectification in response to hyperpolarizing current pulses, and marked AHPs (Figure 3B and Table 2). Depolarized and hyperpolarized phases in cluster 2 neurons occurred at intermediate frequencies, recurring intrinsically with a periodicity of once every ∼500 ms, and were accompanied by a ∼5 mV switching between the bimodal distribution of the membrane potential (Figures 4C and 4D, and Table 3). Morphologically, these neurons had relatively small fusiform somata and medium-sized dendritic arbors (Figure 5B and Table 4).

Cluster 3 neurons were the most prevalent bursting subtype (*n* = 13/24). They exhibited LTS and rebound from hyperpolarization (Figure 3C and Table 2). However, they were excited by NA, indicating a non-sleep-promoting phenotype (Table 2). Their intrinsic electrophysiological properties represented an intermediate between the properties of clusters 1 and 2. Yet, their bursting properties were distinctive: Bursts of AP were long, occurred at low frequencies, recurring with a periodicity of once every ∼2 s, and were accompanied by marked transitions between depolarized and hyperpolarized phase of the membrane potential of ∼9 mV (Figures 4E and 4F, and Table 3).^34^^,^^35^^,^^36^^,^^37^^,^^38^^,^^39^ Morphologically, cluster 3 neurons had medium-sized, ovoid cell bodies and dendritic arbors typically extending parallel to the pia (Figure 5C, and Tables 4 and 5).

**Table 5:** Neurolucida-based dendritic morphology analysis

	1 (*n* = 4)	2 (*n* = 4)	3 (*n* = 7)
(1) Nb of dendrites	4.5 ± 0.2	4 ± 0.4	3.9 ± 0.3
		–	
(2) Dendritic length (μm)	3733.3 ± 405.8	2185.6 ± 654.5	2184.9 ± 266.4
		–	
(3) Dendritic surface (μm^2^)	10118.5 ± 1734.8	5706.1 ± 1507.5	5410.8 ± 693.4
		–	
(4) Dendritic volume (μm^3^)	3112.5 ± 697.4	1685 ± 413	1494.4 ± 236.3
		–	
(5) Length/surface	0.4 ± 0	0.4 ± 0	0.4 ± 0
		–	
(6) Highest order segment	7.3 ± 0.5	6.5 ± 1.4	6.1 ± 0.5
		–	
(7) Dendritic tortuosity	1.4 ± 0	1.4 ± 0	1.3 ± 0
		–	
(8) Nodes	24.8 ± 1.9	18 ± 7.6	13.7 ± 1.7
		–	
(9) Dendritic planar angle	42.9 ± 2	44.6 ± 3.2	42.7 ± 2.5
(10) Dendritic spline angle	47.9 ± 1.9	48.6 ± 4.4	47.1 ± 2.5
		–	
(11) Dendritic sholl (100)	1175.3 ± 224.3	913.3 ± 247.6	803.3 ± 77.8
(12) Dendritic sholl (200)	1166.6 ± 196.9	809.7 ± 358.5	709.5 ± 134.7
		–	
(13) **Dendritic sholl (300)**	**874.8** ± **95.1**	**254.3** ± **124.7**	**343.8** ± **81.2**
**∗**			
(15) Kdim	1.1 ± 0	1.1 ± 0	1.1 ± 0
		–	
(16) Spine 1	13.5 ± 9.7	6 ± 2.9	10 ± 5.2
		–	
(17) Spine 2	18.8 ± 8.4	33.8 ± 12.5	39.9 ± 10.5
		–	
(18) Spine 3	38.3 ± 25.8	49.3 ± 22.8	47.7 ± 8.9
		–	
(19) Spine 4	30.5 ± 24.1	66.5 ± 35.3	33.4 ± 4.5
		–	
(20) Spine 5	16.3 ± 5.3	15.5 ± −10.8	15.3 ± 5.4
		–	
(21) Spine 6	29.3 ± 13.1	7 ± 6	22 ± 9.7
		–	
(22) Spine 7	3 ± 1.2	11 ± 9.8	7.6 ± 5.6
		–	
(23) Spine 8	11.3 ± 8.6	1.8 ± 1.6	0.9 ± 0.6
		–	
(24) Spine density	0 ± 0	0.1 ± 0	0.1 ± 0
		–	

n, number of cells; Kruskal–Wallis test, followed by a Dunn’s post hoc test. Asterisks indicate values significantly different in one cluster vs. all others. Bold type font indicates significance; ∗: significant with p < 0.05.

### Intrinsic membrane oscillations and their propagation to the local network

**Figure 6:**
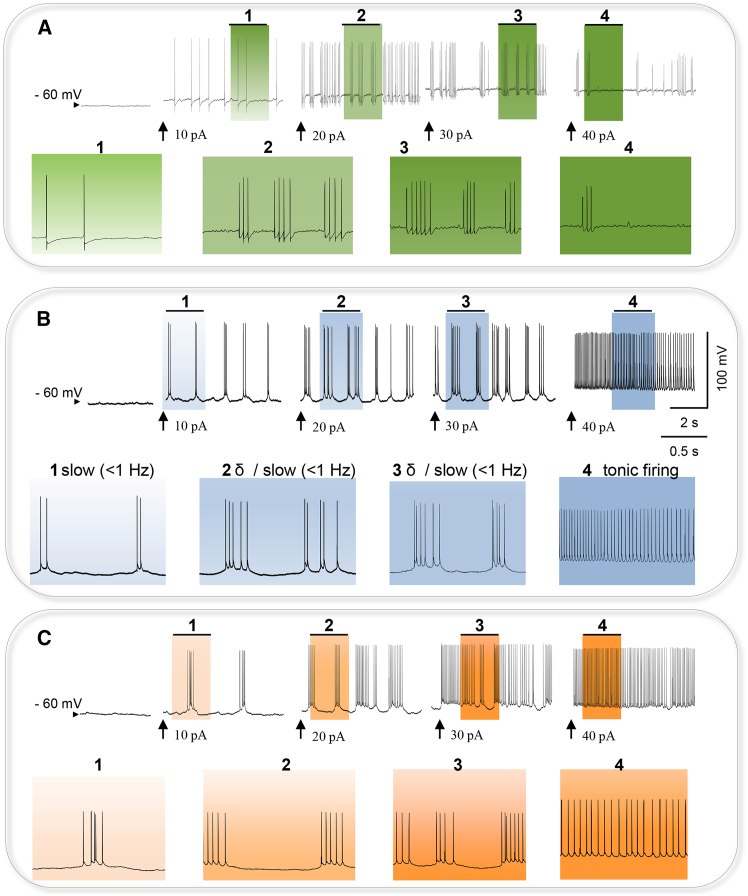
The slow (<1 Hz) oscillation forms part of a continuum of activity in VLPO neurons (A) Whole-cell recording in current-clamp mode of the oscillatory activity. A typical VLPO neuron from cluster 1 is held at different membrane potentials as indicated by arrows. Injections of current pulses progressively led to continuous tonic firing. Sections marked above are expanded below. (B and C) Same as in A, but for neurons from clusters 2 and 3, respectively.

To gain insight into the mechanism underlying bursting, we examined responses to depolarizing current steps (10, 20, 30, and 40 pA; Figure 6). In clusters 2 (*n* = 3) and 3 (*n* = 7), increasing current injections tended to increase the number of AP per burst and the burst frequency, with the largest steps often switching neurons in a state of tonic firing (Figures 6B and 6C). Interestingly, however, cluster 1 neurons tended to maintain a stuttering pattern upon current injection, and the strongest current step tended to reduce overall firing, possibly reflecting saturation (*n* = 5, Figure 6A).

**Figure 7:**
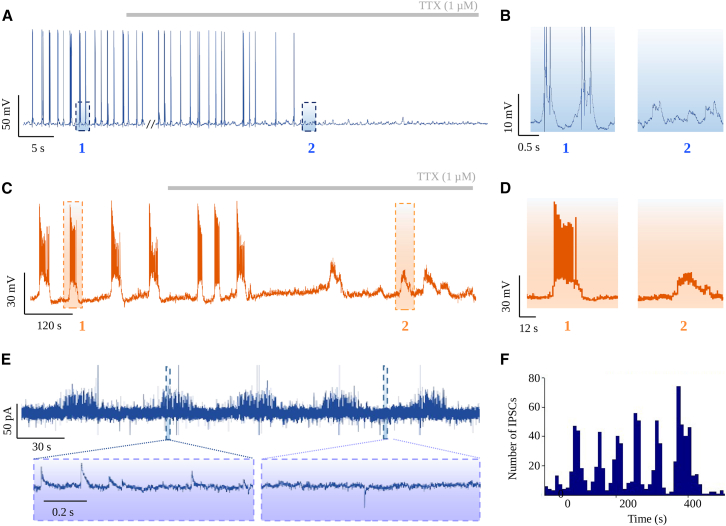
TTX-resistant membrane oscillations and burst-like sIPSCs in VLPO neurons (A–D) Under TTX, neurons from clusters 2 (*n* = 2) and 3 (*n* = 5) continue to exhibit spontaneous membrane-potential oscillations. Inset: High magnification of the depolarized phase indicated by a red dotted rectangle. (E) In 4 out of 66 neurons, recordings of spontaneous inhibitory postsynaptic currents (sIPSCs) in the voltage clamp configuration in the absence of TTX revealed burst-like episodes. Left: intraburst epoch (red dotted rectangle; high sIPSC frequency). Right: interburst epoch (red dotted rectangle; low sIPSC frequency). (F) Histogram of sIPSC event counts for the recording shown in (E).

We next tested the effect of TTX on spontaneous oscillatory activity. As expected, TTX abolished spiking in all neurons. Interestingly, however, oscillations of the membrane potential persisted in all neurons except for 2 neurons from cluster 3 (Figures 7A–7D). This indicates that, in most cases, oscillations of the membrane potential do not require Na^+^-dependent spiking and is compatible with an intrinsic oscillatory mechanism. In the remaining 2 neurons from cluster 3, TTX completely abolished the oscillatory depolarized and hyperpolarized phases of the membrane potential, suggesting a Na^+^-dependent mechanism underlying the oscillation in these cells. This dependence may reflect AP–driven synaptic inputs and/or intrinsic sodium conductances contributing to burst generation.

To evaluate a synaptic contribution more directly, we examined voltage-clamp recordings of spontaneous excitatory and inhibitory postsynaptic currents (sEPSCs and sIPSCs) obtained in 66 neurons out of 289 whole-cell recordings.^12^ We identified 4 neurons that presented rhythmically clustered bursts of IPSCs (Figures 7E and 7F). Intraburst sIPSCs frequency (2.75 ± 0.36 Hz) was significantly higher than the interburst frequency of (0.30 ± 0.09 Hz; *n* = 4; *p* < 0.029; Mann-Whitney *U*-test). All these neurons were NA(−), and 3 out of 4 were endowed with an LTS. The combined observation of UP and DOWN-state and bursts of sIPSCs in neighboring neurons suggests that intrinsically bursting neurons can propagate slow rhythmic activity to NA(−) sleep-promoting neurons of the VLPO through inhibitory synaptic inputs.

## Discussion

Here, we report that VLPO neurons can exhibit intrinsically generated rhythmic bursting activity. Spontaneous bursting was screened in a database of 393 *ex vivo* whole-cell and loose cell-attached recordings and occurred in an estimated 12.47% of VLPO neurons. Analyses of loose-patch recordings revealed bursting in both NA(−) putative sleep-promoting neurons and NA(+) putative wake-active neurons. Unsupervised clustering performed on 24 whole-cell recordings confirmed that bursting behavior in the VLPO occurs in NA(−) putative sleep-promoting neurons and discriminated two NA(+) putative wake-active neuron subtypes, respectively displaying fast and slow bursting dynamics. Interestingly, oscillations of the membrane potential persist in the presence of TTX in most tested neurons, indicating that the underlying rhythm is predominantly intrinsic rather than synaptically driven. Collectively, these findings suggest that the VLPO may contribute to slow rhythmic dynamics within sleep-wake networks, although its role in large-scale oscillations remains to be established.

Rhythmic bursting activity in VLPO neurons has not been previously described. The lack of a previous report likely reflects the low probability of observing spontaneous bursting in *ex vivo* slices and the need for large datasets. Here, the study of spontaneous bursting was conducted in parallel with other studies of the properties of VLPO neurons,^11^^,^^12^^,^^23^^,^^30^ making it possible to sample a large dataset.

### Identity of bursting VLPO neurons

Our clusters were defined based on intrinsic electrophysiological properties, bursting parameters, morphological features, and responses to noradrenalin application, with bursting-related dynamics providing the primary discriminative parameter. While we did not directly assess the major neurotransmitter released by clustered neurons, the existing literature allows cautious speculation regarding their potential neurochemical identity. The NA(−) cluster (cluster 2) is consistent with the canonical sleep-promoting VLPO population, classically described as predominantly GABAergic/galaninergic and inhibited by monoamines.^9^^,^^10^^,^^13^^,^^14^^,^^17^^,^^18^^,^^19^ On the other hand, the two NA(+) clusters (clusters 1 and 3) likely correspond to wake-active VLPO neurons. It is conceivable that one could represent a wake-active GABAergic subtype, potentially reflecting the overall predominance of inhibitory neurons in this region, and therefore Cluster 3 neurons, which are the most numerous,^6^^,^^9^^,^^10^^,^^11^^,^^12^ whereas Cluster 1 neurons could correspond to the glutamatergic wake-promoting neurons recently described in the preoptic/VLPO region.^25^^,^^26^^,^^27^ However, definitive assignment of transmitter phenotype will require future studies combining electrophysiology with molecular or genetic identification.

### Cluster-specific mechanisms

Understanding the function of rhythmic bursting in the VLPO might require identifying and modulating the ion conductance involved in its initiation and maintenance. It is worth noting that rhythmic bursting presents distinct temporal characteristics in neurons from clusters 1 and 3, suggesting that it may rely on distinct mechanisms in these two groups. Accordingly, while current injection tended to abolish bursting and induce continuous firing in neurons from clusters 2 and 3, bursting was maintained in neurons from cluster 1. Cluster 1 Na(+) and stuttering neurons tend to maintain a relatively depolarized state, and their bursting appeared to result from failures of spike initiation (Figures 3A and 6A). This pattern is reminiscent of a “stuttering” firing, which has been described in cortical interneurons and depends on D-type K^+^ currents.^40^^,^^41^^,^^42^ In clusters 2 and 3, however, bursting seems to rely on intrinsically generated oscillations of the membrane potential (Figures 3B, 3C, 6B and 6C). Such intrinsic oscillatory dynamics have been described in cortical layer^38^^,^^39^^,^^43^ and 6b,^44^ shown to depend on persistent non-inactivating voltage sodium currents.^45^ However, such conductance is TTX-sensitive.^46^ Thus, VLPO membrane potential oscillations likely depend on TTX-insensitive conductance acting similarly. Interestingly, neurons from clusters 2 and 3 displayed LTS, which is thought to depend on T-type calcium currents.^28^^,^^29^^,^^47^ These currents are capable of inducing spontaneous depolarization of the membrane potential.^48^^,^^49^ Rhythmic oscillations may require an interplay between inward drive and mechanisms that promote repolarization necessary for the reactivation of T-type calcium channels.^28^^,^^29^ As the depolarization phase does not rely on TTX-sensitive voltage-gated Na^+^ channels, the hyperpolarization phase is also unlikely to depend on Na^+^-activated K^+^ currents.^50^^,^^51^^,^^52^ Instead, Ca^2+^ activated K^+^ currents may contribute to repolarization,^53^ together with inactivation of the depolarizing conductance. These mechanisms are not mutually exclusive.

### Intrinsic oscillation dynamics and extrinsic network mechanisms

We observed that in most tested neurons, slow depolarized/hyperpolarized oscillations of the membrane potential persisted in TTX, indicating an intrinsic generator. Consistently, we occasionally observed rhythmically modulated GABAergic inputs to putative sleep-promoting VLPO neurons recorded in voltage clamp. These inhibitory events occurred at the same rhythm as the intrinsic oscillation. A parsimonious interpretation is that intrinsically oscillating VLPO neurons rhythmically inhibit nearby neurons, possibly sharpening or amplifying slow bursting through phase-locked IPSCs.

Gap-junctions are particularly effective at synchronizing inhibitory networks,^54^^,^^55^ and may also contribute to shape VLPO firing patterns, as proposed in the thalamic oscillatory circuits.^35^ In addition, dynamic neuron-glia interactions could further tune local excitability and timing, as described in the olfactory bulb.^56^ Moreover, in sleep-promoting VLPO neurons, metabolic control via K_ATP channels may gate burst permissiveness by adjusting baseline excitability.^24^ Together with the phase-locked GABAergic inputs observed in NA(−) neurons, these elements suggest a hybrid model in which intrinsic oscillators provide the depolarized state drive, while local inhibitory coupling and potential reinforced gap junctions or astroglial mechanisms, phase-align and amplify rhythmic activity within VLPO circuits.

### Circadian and developmental modulation of VLPO rhythmicity

Beyond local circuit pacing, global state variables such as circadian phase may also tune baseline excitability. Because all recordings were performed during the early light phase, corresponding to the beginning of the rest period in nocturnal rodents, circadian timing may have influenced baseline excitability and bursting propensity. In addition, recordings were obtained from juvenile mice (P14–P21), a period during which sleep–wake regulation, neuromodulatory tone, and intrinsic membrane properties are still maturing, potentially affecting both excitability and the likelihood of observing spontaneous rhythmic bursting *ex vivo*.

It is also well established that neuronal intrinsic properties and firing rates can vary across circadian time, in hypothalamic and arousal-related nuclei, reflecting daily modulation of membrane conductances and synaptic tone.^57^^,^^58^^,^^59^^,^^60^ Circadian phase and developmental stage could therefore influence the basal activity levels and the properties of particular functional phenotypes.

### Generalization across the hypothalamus

Interestingly, our results are reminiscent of data obtained in other regions of the hypothalamus. Robust evoked bursts of mIPSCs were indeed also recorded in hypothalamic magnocellular neurons.^61^ In contrast, rhythmically clustered bursts of EPSPs have been described in oxytocin neurons in organotypic hypothalamic cultures.^62^ These pieces of evidence suggest that the ability to produce rhythmic bursting is widespread in the hypothalamus and that the hypothalamic network might shape the rhythmically clustered bursts firing on putative sleep-promoting neurons through bursting afferent volleys of IPSCs. Overall, bursting activity in the VLPO is likely governed by multiple interacting mechanisms, as in other brain regions.^38^^,^^39^^,^^43^^,^^45^ Future work will thus have to disentangle these mechanisms to clarify the role of bursting activity in VLPO neurons.

### *In vivo* relevance

Burst firing has been reported *in vivo,* in sleep-active VLPO neurons, with extracellular recordings revealing clustered discharge during sleep.^22^^,^^63^^,^^64^^,^^65^^,^^66^ Similar state-dependent firing has also been described in wake-active populations.^67^^,^^68^ However, because these studies relied on extracellular recordings, the underlying membrane potential dynamics could not be assessed, and a slow, cell-autonomous membrane potential oscillation of the type described here has not been explicitly demonstrated *in vivo*. Burst firing could therefore reflect an intrinsic rhythmic mechanism, but it may also arise from network-driven fluctuations. Moreover, whether VLPO neurons burst synchronously or maintain coordinated rhythmicity over time remains unknown. Indeed, burst frequency in hypothalamic neurons can vary considerably across neurons and within individual cells during prolonged recordings,^63^ suggesting a flexible modulation rather than a fixed pacemaker rhythm.

The firing of sleep-active VLPO neurons is known to be modulated by sleep pressure,^65^ intrinsic factors such as adenosine,^10^^,^^22^^,^^69^ and synaptic inputs from interconnected regions.^8^^,^^70^ Within this modulatory framework, intrinsic oscillations could shape VLPO burst firing and its dynamic entrainment during sleep. Such oscillations may act as a local coordinating signal, rhythmically inhibiting neighboring NA(−) neurons and promoting clustered GABA/galanin output from the VLPO, thereby imposing a rhythmic inhibition on downstream wake-promoting centers such as the LC, TMN, and LH. The infraslow NA/LC fluctuations reported during NREM sleep by Lüthi and colleagues may reflect a periodic disinhibition of the LC driven by rhythmic inhibition within the VLPO.^71^ However, intrinsic VLPO oscillations should not be interpreted as direct generators of cortical slow waves. Instead, they may represent a local timing mechanism that organizes burst discharge and inhibitory coupling within VLPO circuits, contributing to the temporal structuring of sleep-promoting output rather than directly driving large-scale cortical rhythms.

Future *in vivo* recordings and causal manipulations of VLPO rhythmicity will be critical to determine whether these intrinsic oscillations represent a local microcircuit mechanism or a distributed pacemaker signal in the regulation of sleep dynamics.

### Limitations of the study

While this study provides the first characterization of intrinsic rhythmic bursting in VLPO neurons, several limitations should be acknowledged. First, all recordings were performed in acute *ex vivo* slices from juvenile mice (P14–P21), a developmental window during which sleep–wake circuitry, neuromodulatory tone, and intrinsic membrane conductances are still maturing; whether the bursting phenotypes described here persist with the same prevalence and properties in adult animals, therefore, remains to be determined. Second, the unsupervised clustering analysis was performed on a relatively modest sample of whole-cell recordings (*n* = 24), which may limit the statistical robustness of the three-cluster solution, particularly given the proximity between clusters 2 and 3 revealed by silhouette analysis. Third, while TTX experiments support an intrinsic oscillatory mechanism in most tested neurons, a contribution of electrotonic coupling via gap junctions or residual network activity in the slice cannot be fully excluded. Finally, the functional relevance of these intrinsic oscillations for sleep–wake behavior *in vivo* remains to be established, and causal manipulations such as optogenetic silencing or activation of identified bursting VLPO populations during polysomnographic recordings will be critical to determine whether this local rhythmic mechanism contributes to the temporal organization of sleep.

## Resource availability

### Lead contact

Further information and requests for resources and reagents should be directed to and will be fulfilled by the lead contact, Armelle Rancillac (armelle.rancillac@college-de-france.fr).

### Materials availability

This study did not generate new, unique reagents.

### Data and code availability

• All data in this paper will be shared by the lead contact upon request.
• This paper does report original code as indicated in the STAR Methods.

## Acknowledgments

This work was supported by the 10.13039/501100004794Centre National de la Recherche Scientifique (CNRS), the French Institute of Health and Medical Research (10.13039/501100001677Inserm). We thank all members of the animal house facility at 10.13039/501100003068ESPCI.

## Author contributions

Conceptualization A.R. and methodology, Q.P., J.R., and A.R.; formal analysis, Q.P., J.R., and A.R.; investigation, H.G., T.G., A.R.; writing – review and editing, Q.P, A.R.; Critically revised the manuscript, all authors; supervision and project administration, A.R.

## Declaration of interests

The authors declare no competing financial interests. Non-financial disclosure: none.

## Declaration of generative AI and AI-assisted technologies in the writing process

During the preparation of this work, the authors used AI to improve grammar and English writing. After using this tool/service, the authors did not accept all the suggested changes and reviewed the content as needed and take full responsibility for the content of the published article.

## STAR★Methods

### Key resources table

**Table:** 

REAGENT or RESOURCE	SOURCE	IDENTIFIER
**Chemicals, peptides, and recombinant proteins**		

Sigma Aldrich - Noradrenalin	N/A	108341-18-0
Latoxan - TTX	N/A	L85 03
Sigma Aldrich - Biocytin	N/A	576-19-2
Roche - DAB	N/A	11718096001
Sigma Aldrich - MOWIOL	N/A	243.011773.47

**Deposited data**		

Slow Intrinsic Oscillations in the Ventrolateral Preoptic Nucleus - Dataset	figshare.com	https://doi.org/10.6084/m9.figshare.31647832

**Experimental equipment**		

Two-photon microscope	Zeiss	N/A
Confocal microscope	Yokogawa spinning-disk	N/A
Upright Axioskop	Zeiss	N/A

**Experimental models: Organisms/strains**		

Mouse: C57BL/6J	Charles River	N/A

**Software and algorithms**		

Microsoft Office – PowerPoint, Excel	Commercially Available Software	N/A
Mathwork - MATLAB	Commercially Available Software	N/A
Axon Instruments - pCLAMP	Commercially Available Software	N/A
MBF Bioscience - Neurolucida Explorer	Commercially Available Software	N/A
Media Cybernetics Inc.- Image-Pro 7 software	Commercially Available Software	N/A
GraphPad - Prism	Commercially Available Software	N/A

### Experimental model and study participant details

#### Study design and ethics

This study was received ethical approval from the European Community Council Directive of 22 September 2010 (010/63/UE) and the local ethics committee (Comité d’éthique en matière d’expérimentation animale number 59, C2EA—59, “Paris Center et Sud”). The number of animals in our study was accordingly kept to the necessary minimum.

#### Animals

C57BL/6J juvenile male mice (14–21 days old; Charles River, France) were housed in a temperature-controlled (20°C–22°C) room under a 12–hour light-dark cycle (lights on at 09:00 a.m.) with *ad libitum* access to food and water and acclimated in the laboratory for at least 1 week before experiments.

### Method details

#### Preparation of acute hypothalamic slices

Animals were decapitated at the beginning of the light phase, between 09:00 and 10:00 a.m. Brains were quickly extracted and submerged in ice-cold artificial cerebrospinal fluid (aCSF (mM): 130 NaCl; 5 KCl; 2.4 CaCl_2_; 20 NaHCO_3_; 1.25 KH_2_PO_4_; 1.3 MgSO_4_; 10 D-glucose; 15 sucrose, pH = 7.35) containing the glutamate receptor blocker kynurenic acid (1 mM) and constantly oxygenated with 95% O_2_–5% CO_2_. Coronal brain slices (300 μm thick) were cut with a vibratome (VT2000S; Leica) and stored in oxygenated aCSF containing kynurenic acid until further use.

#### Loose patch and whole-cell patch-clamp recordings

Individual slices were placed on the stage of an upright microscope (Zeiss) in a recording chamber maintained at 32°C and continuously superfused with oxygenated artificial aCSF. Slices were visualized under infrared light with Dodt gradient contrast (Luigs & Neumann) through a 40× immersion objective and a CCD camera (Hamamatsu). Electrophysiological traces were amplified with a MultiClamp700B amplifier (Axon Instruments) and digitized by an acquisition board (Digidata 1440; Axon Instruments) connected to a computer running pCLAMP (Axon Instruments). Recordings were targeted to the VLPO and performed in the whole-cell and loose cell-attached configurations with patch-clamp pipettes (3–6 MΩ) filled with an internal solution containing (in mM): 144 K-gluconate; 1 MgCl_2_; 0.5 EGTA; 10 HEPES (pH 7.2) and 2 mg/mL biocytin (Sigma Aldrich) for morphological analysis. The osmolarity of the internal solution was adjusted to 285–295 mOsm. The liquid junction potential between the patch pipette and the perfused extracellular solution was estimated at 11 mV and was not corrected.

Spontaneous currents were recorded in voltage-clamp mode at a holding potential of −60 mV, which is more depolarized than the reversal potential for inhibitory postsynaptic currents (IPSCs). The pipette solution contained a low chloride concentration (1 mM), resulting in an E_Cl_ of −111 mV. Therefore, inward currents were identified as spontaneous excitatory postsynaptic currents (sEPSCs) and outward currents as sIPSCs. Signals were filtered at 2 kHz, digitized at 10 kHz and acquired online using the pCLAMP 9 (Clampex; Axon Instruments).

#### Drugs delivery

Noradrenaline (NA, 10 μM; Sigma) or Tetrodotoxin (TTX, 1 μM, Sigma) was mixed in aCSF and applied to slices via superfusion for 30 s. Drug application was preceded and followed by superfusion with regular aCSF.

#### Histology

After recordings, slices were immersed overnight in 0.1% phosphate buffer (PB) adjusted to pH 7.4 containing 4% paraformaldehyde and stored in PB at 4°C until further use. Biocytin was revealed through diaminobenzidine staining (DAB, Vector) performed after binding of avidin-horseradish peroxidase through kit ABC (Thermofisher). Slices were mounted in MOWIOL.

#### Burst analysis

Action potentials were detected using the template search function of Clampfit (pCLAMP10, Molecular Probes). Bursts of action potentials were then identified using the Clampfit “Poisson Surprise” protocol^72^ and characterized by the following parameters. The average (1) depolarized phase and (2) hyperpolarized phase membrane potential were measured, and (3) delta was taken as their difference. For the Depolarized Phase and Hyperpolarized Phase, values were computed from the underlying baseline membrane potential, with action potentials manually excluded so they were not included in these averages.

Spike train autocorrelograms were computed for each recorded neuron using a symmetrical time window ranging from −2500 ms to +2500 ms with 20 ms bin resolution. To avoid contamination by the large peak around 0 ms corresponding to the refractory period and immediate spike correlations, the central window from −250 ms to +250 ms was systematically excluded from further analysis. To quantify rhythmicity, a sine wave of the form y(t) = a sin(2πf t) + b, where a is the oscillation amplitude, f the frequency, and b the vertical offset, was fitted to the remaining autocorrelogram data using a least-squares optimization procedure. (4) The rhythmicity index was defined as the **ratio a/b**, where higher values reflect stronger oscillatory modulation relative to baseline autocorrelogram level, and (5) the frequency f = 1/T corresponds to the dominant rhythmic firing frequency (respectively period T) of the neuron. (6) Rise time and (7) decay time were measured in Clampfit with cursors manually placed at the beginning and end of state transitions and averaged over bursts. Likewise, (8) burst duration and (9) number of action potentials per burst were averaged over bursts for each recording. (10) Intra-burst frequency and (11) inter-burst frequency were the number of action potentials per unit of time during and outside of bursts, respectively. (12) The overall firing rate and (13) mean inter-burst intervals were also measured.

To facilitate comparison across previously published datasets, we summarized the number of neurons recorded in loose-patch and whole-cell configurations and classified them according to their responsiveness to noradrenaline (NA−, NA+, or not tested) (Table 6).

**Table 6:** Distribution of neurons recorded in loose-patch and whole-cell configurations across studies, according to noradrenaline responsiveness

	Loose Patch			Whole-cell			
**Article**	NA-	NA+	Not tested	NA-	NA+	Not tested	**Total**
Varin et al., 2015	7					8	**15**
Sangare et al., 2016	14			56			**70**
Scharbarg et al., 2016	17	16					**33**
Dubourget et al., 2016				79	110	100	**289**
Total							**393**

Numbers correspond to neurons reported in each study and were extracted from the original publications. Neurons were classified as non-responsive (NA−), responsive (NA+), or not tested following noradrenaline application. Bold type font indicates the total number of neurons included from contributing studies and overall.

#### Electrophysiological properties

In whole-cell recordings, 27 electrophysiological parameters were measured from the voltage responses induced by 800 ms current steps, ranging from −100 pA to firing saturation in 10 pA increments, while neurons were at a membrane potential of −60 mV.^73^^,^^74^ (1) The Resting Membrane Potential (RMP) was measured immediately after achieving whole-cell configuration. (2) Input resistance (Rm) was estimated from the voltage deflection elicited by −10 pA hyperpolarizing current using Ohm’s law (R = U/I). (3) Membrane time constant (t_IC_) was measured on the same trace as the time constant of an exponential fitted to the response onset. (4) The membrane capacitance (Cm) was calculated according to the equation Cm = t_IC_/Rm. Under our conditions, injection of hyperpolarizing current pulses often induced a hyperpolarization-activated cationic current (Ih) that followed the initial hyperpolarization peak, known as a sag. (5) R_hyp_ and (6) R_sag_ were measured as the slope of the linear portion of an I–V plot measured at the beginning (0–0.1 s; −100 to 0 pA) and at the end of hyperpolarizing current pulses (0.7–0.8 s; −100 to 0 pA). (7) ΔG_Sag_ corresponds to (R_sag_–R_hyp_)/R_sag_. A distinctive electrophysiological feature of sleep-promoting VLPO neurons is the presence of Low Threshold Spikes (LTS), defined as T-type Ca^2+^-mediated rebond depolarization that occurs upon release from sufficiently strong hyperpolarization and can support a burst of action potentials. In our dataset, two rebound-related features were encoded as binary variables: (8) post-inhibitory rebond, defined as a rebound depolarization (with or without action potential firing) evoked by a 100 pA hyperpolarizing current step delivered from −60 mV; and (9) LTS, defined as a rebound depolarization with associated spike elicited occurring upon termination of a −100 pA hyperpolarizing current pulse that drove the membrane potential below −80 mV. (10) Rheobase was the minimal depolarizing current amplitude eliciting at least one action potential during a fixed-duration current step (800 ms) from resting membrane potential. (11) The first spike latency was the time needed to elicit a spike at rheobase from current injection onset. Neurons can exhibit bursting, adapting, regular, or stuttering firing behavior in response to depolarizing currents. To capture this diversity, the instantaneous firing frequency (1/inter-spike intervals) was measured in response to the minimal current injection eliciting more than three action potentials and fitted with a linear curve. (12) Adaptation (m_threshold_) and (13) minimal steady state frequency (F_threshold_) were taken respectively as the slope and the intersect of this linear fit. Instantaneous firing frequency was then measured on the maximal current step before saturation and fitted with the sum of an exponential and a linear function according to the equation F_sat_ = A_sat_ × E^−t/τsat^ +t × m_sat_ + F_offset_, where (14) A_sat_ corresponds to the initial amplitude of the exponential adaptation, (15) t_sat_ to the time constant of the exponential decay fitting early firing rate adaptation, (16) F__offset_ the maximal steady-state frequency, and (17) m_sat_ to the slope of the linear component accounting for the slow change in firing rate over time and t to the time. (18 and 24). Amplitudes of first (A1) and second (A2) action potentials were measured from spike onset in the train induced by the minimal current step evoking at least 3 action potentials, and (19 and 25) spike durations (D1 and D2) were measured on the same spikes at half-amplitude. (20) The amplitude (AHP_max_) and (21) time (tAHP_max_) of after-hyperpolarization were measured relative to spike onset on the first action potential. When AHPs presented a biphasic shape, (22) the amplitude (ADP) and (23) time (tADP) of the rebound (i.e., the so-called After Depolarization Potential) were measured from the first hyperpolarization peak. (26) Amplitude variation (Var A) and (27) duration variation (Var D) were calculated as (A1-A2)/A1∗100 and (D2-D1)/D1∗100, respectively. All neurons responded to NA. In addition to intrinsic electrophysiological properties (28), the hyperpolarizing or depolarizing pharmacological response to NA application was recorded in current-clamp mode and encoded as either 0 or 1, respectively, by a binary variable.

#### Morphological analysis

Neuron morphology was quantified with 10 parameters related to the features of somata and 24 parameters related to those of dendrites. Morphologies of somata were reconstructed from infrared images taken before whole-cell recording using Image-Pro 7 software (Media Cybernetics Inc.). Morphological features were measured from these images after calibration with a standard 24 μm grid. The soma contour was manually drawn from the image, and (1) the cell body area and (2) perimeter were measured. (3) The form factor was defined as the ratio between the perimeter and the area. The (4) maximal diameter and (5) minimal diameter passing through the centroid were measured, and (6) the aspect ratio was taken as the maximal over the minimal diameter. (7) The somatic solidity was defined as the ratio of the somata area over its convex area. Likewise, (8) convexity was taken as the ratio of the convex perimeter over the perimeter. The measurement of how closely this shape approached that of a circle was assessed throughout (9) the roundness. Finally, (10) somatic compactness was defined as √(4/π) × area/D_feret_max_ where D_feret_max_ is the maximal feret diameter (i.e., the diameter as if measured by calipers).

The dendritic properties were measured from 3-dimensional reconstructions of biocytin-filled arborization performed using the Neurolucida platform. Parameters were extracted using Neurolucida Explorer (MBF Bioscience) and Excel (Microsoft). To be able to describe the differences of dendritic arborizations, we systematically quantified the following parameters: (1) Number of primary dendrites, (2) total dendritic length, (3) total dendritic surface, (4) total dendritic volume, (5) ratio of the dendritic length over dendritic surface, (6) highest dendritic order segment, (7) average tortuosity of dendritic segments (defined as the ratio of the length of the segment over the straight line path between its extremities), (8) number of dendritic nodes and (9) dendritic planar angle. We performed a Sholl analysis^75^ to describe differences between neuronal arborization. (10) The length of the dendritic arborization that could be enclosed within a 100 μm radius circle around the cell body, (11) between 100 μm and 200 μm radii, (12) between 200 μm and 300 μm radii, and (13) outside a 300 μm radius was systematically extracted. (14) Kdim, a measure from fractal analyses, represented the degree to which the dendritic arbor has a scale-invariant topology. Finally, (15–23) the number of dendritic spines in segment order, ranging from 1 to 8, was extracted, and (24) mean spine density was calculated.

#### Ward’s clustering and silhouette

Unsupervised clustering of bursting neurons was performed in the MATLAB environment (Mathwork) as described previously^12^^,^^74^^,^^76^^,^^77^ and was based on a total of 34 parameters: 9 burst parameters: Depolarized Phase, Hyperpolarized Phase, Delta, a/b, Rise Time, Decay Time, Burst duration, Burst Frequency and Interevent interval; 2 morphological parameters: cell body area and perimeter and 2 binary electrophysiological parameters: LTS, Rebond, 7 passive electrophysiological parameters, RM, Rm, tIC, Cm, Rhyp, Rsag, ΔG_Sag_, 6 firing electrophysiological parameters: Rheobase, 1st spike delay, Asat, tsat, Csat, 8 spike parameters: A1, D1, AHP max, ADP1, tAHP, tADP1, Var A, Var D, and responsiveness to noradrenaline: NA. Clustering was also performed while excluding or selectively including each parameter group to assess their impact on clustering results.

Briefly, the parameters were z-scored, and a dendrogram of neuron’s proximity in the parameter space was constructed with Ward’s method^31^ based on Euclidean distances. Clusters generated by Ward’s method were further refined using the K-means algorithm initialized on Ward’s cluster centroid to ensure that clusters did not overlap. The quality of a clustering was assessed to determine the optimal clustering number using silhouette analysis^78^ and the normalized sum of squared errors (SSE).^79^

Silhouette value was defined for each neuron *i* as:

$$s\left(i\right)=\frac{a\left(i\right)-b\left(i\right)}{\max\{a\left(i\right)\;;\;b\left(i\right)\}}$$

Where *a(i)* is the average distance of neuron *i* to the neurons from its cluster, and *b(i)* is the average distance to the neurons of the closest cluster. A positive silhouette value indicates that, on average, the neuron is closer to the neurons of its own cluster than to the neurons belonging to the closest clusters. On the contrary, a negative value indicates a potential misclassification.

SSE was defined as:

$$SSE=\sum_{i=1}^{k}\sum_{x_{j}\in C_{i}}{\left\|y_{j}-c_{i}\right\|}^{2}$$

Where *y*_*j*_ is the *j*th object in cluster *C*_*i*_ and *c*_*i*_ the center of cluster *C*_*i*_. SSE naturally decreases when cluster number (i.e., the number of model parameters) increases. To evaluate what cluster number best minimizes SSE relative to expected decreases, we estimated the distribution of SSE values for each cluster number by performing clustering on shuffled data matrices where parameter values were randomly reassigned between neurons (i.e., neurons). Shuffling disrupts the correlation structure of the data matrix without changing parameter distribution. Normalized SSE was then computed as:

$$Z\left(SSE\right)=\left(SSE-\mu_{SSE}\right)/{\sigma \mu }_{SSE}$$

Where μ_SSE_ and σ_SSE_ are the mean and standard deviation of SSE values obtained by random shuffling.^79^ Shuffling was performed 5000 times.

### Quantitative and statistical analysis

All data are expressed as the mean ± standard error of the mean (SEM). Statistical differences between the three clusters were determined using the Kruskal-Wallis one-way nonparametric ANOVA using Prism (GraphPad software, version 8, CA USA). *p*-values of ≤0.05 were considered statistically significant. In all cases, n refers to the number of examined neurons.
