# Supplementary material for: Slow intrinsic oscillations in the ventrolateral preoptic nucleus
Source: iScience. 2026 Jul 3;29(7):116477. doi: 10.1016/j.isci.2026.116477 (PMC13355658; doi:10.1016/j.isci.2026.116477)
Supplement: Document S1. Figures S1–S3 [file mmc1.pdf]

iScience, Volume *29*

Supplemental information

Slow intrinsic oscillations

in the ventrolateral preoptic nucleus

Quentin Perrenoud, Jérôme Ribot, Hélène Geoffroy, Thierry Gallopin, Nathalie Rouach, and Armelle Rancillac

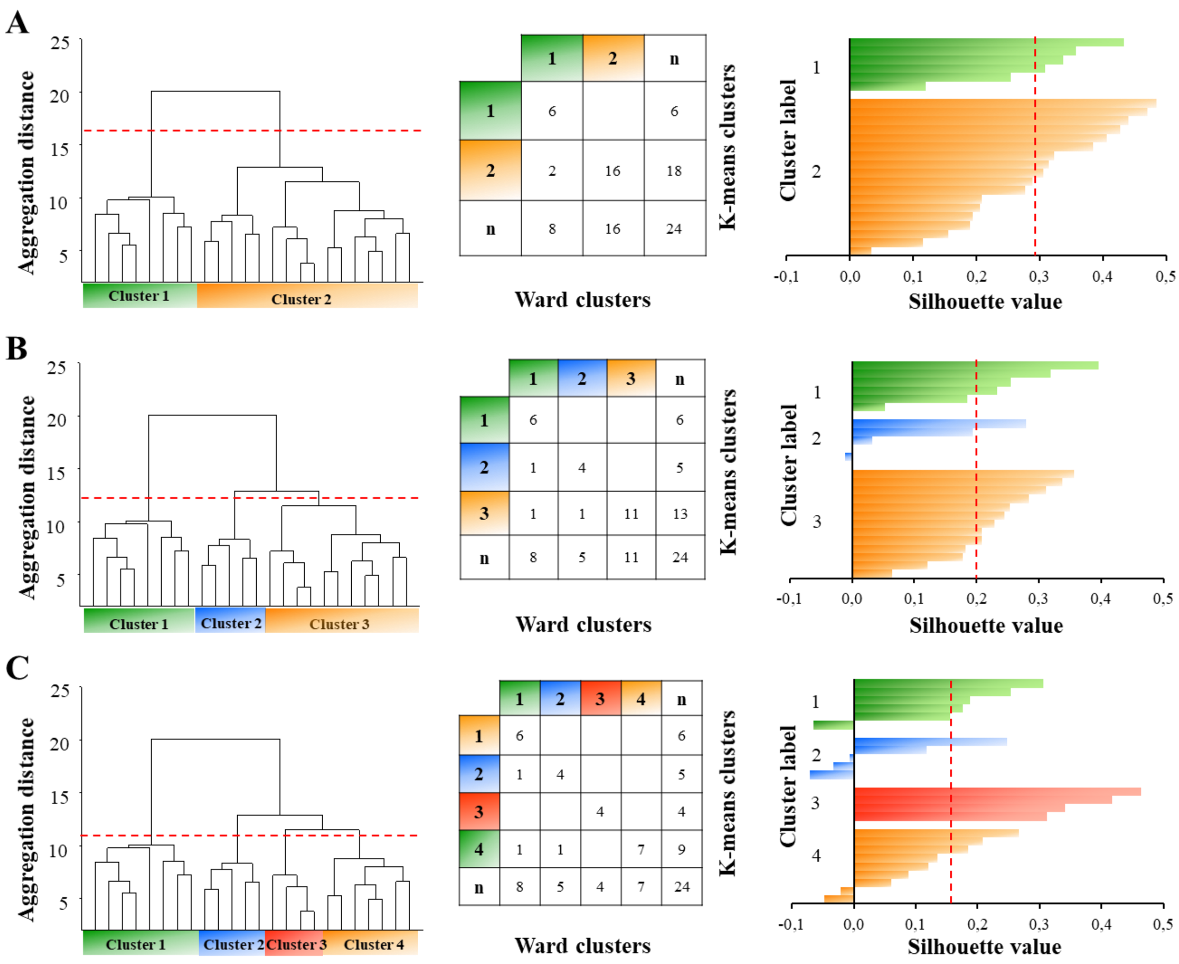

Figure S1. Unsupervised clustering of 24 bursting VLPO neurons

(A) Ward's clustering of 24 bursting neurons. Individual cells are represented along the x-axis. Same as in Fig. 2, with only two clusters: 1 (in green) and 2 (in orange) (*left*). Clusters generated by Ward's method in (A) were corrected using the clustering output generated by the *K-means* algorithm (*middle*). The silhouette analysis was performed to assess the quality of the clustering (mean value indicated by the red dashed line).

(B) Same as in A, with 3 clusters.

(C) Same as in A, with 4 clusters.

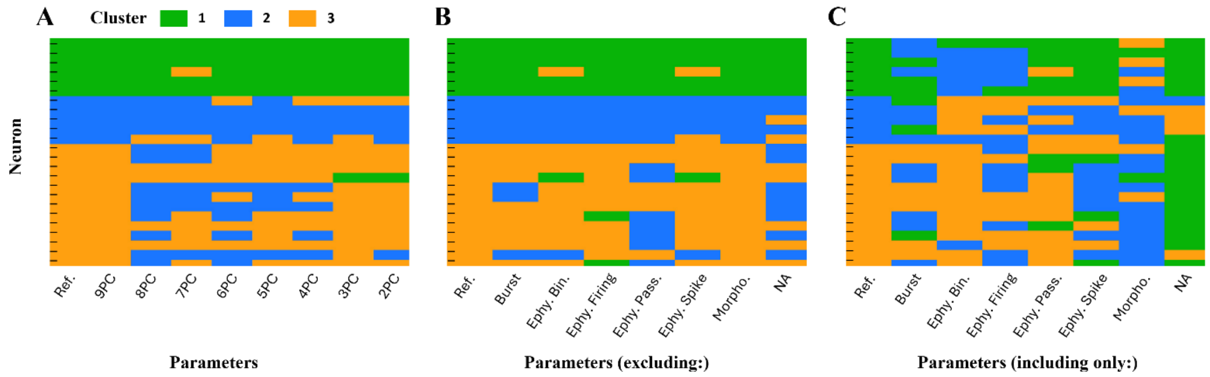

Figure S2. Parameter dependency of unsupervised clustering

(A) Cluster assignment of neurons (rows) when considering 9 to 2 principal components (columns). Reference cluster assignment is shown in the leftmost columns (Ref.). The cluster structure is mostly conserved when considering a low number of PCs.

(B) Same as in A when excluding selected groups of parameters: burst (Burst), binary electrophysiological (Ephy. Bin.), firing electrophysiological (Ephy. Firing), passive electrophysiological (Ephy. Pass.), spike (Ephy. Spike), morphological (Morpho.), and noradrenaline (NA). Excluding passive electrophysiological parameters or NA disrupts the separation between clusters 2 and 3.

(C) Same as in B, when selectively including groups of parameters and excluding all others.

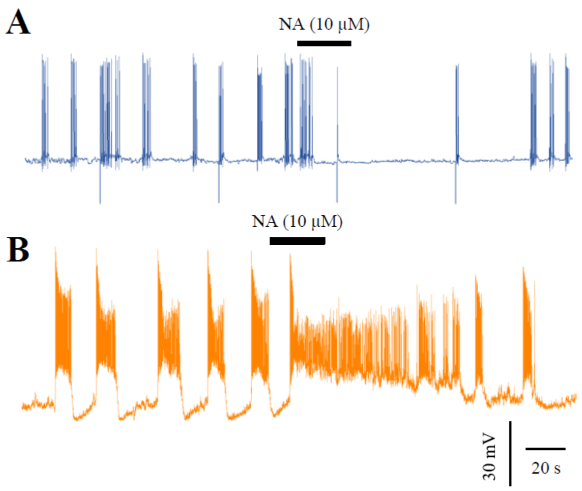

Figure S3. Pharmacological identification of sleep-promoting neurons

(A) inhibitory effect of bath-applied (30 s) noradrenaline (NA, 10 µM).

(B) excitatory effect of bath-applied (30 s) noradrenaline (NA, 10 µM).
